# Medical Opinion and Movement

**Published:** 1910-01-01

**Authors:** 


### Medical Opinion and Movement.

PROFESSOR HELLER, of Kiel, advocates the local application of a 2.5 per cent. solution of chloral hydrate for all manner of Inflammatory Conditions of the Throat and Mouth. It is strongly antiseptic and parasitic, but it also acts as a local anæsthetic and at the same time excites an intense hyperæmia and transudation. He considers it greatly superior to other more usual applications, especially chlorate of potassium. A small quantity of the solution should be taken into the mouth and thrown about by masticatory movements and by movements of the head from one side to the other, but not gargled. This should be repeated every half hour. It is particularly efficacious in inflammatory conditions involving the tonsils. In stomatitis of mercurial origin a spray of a 2 per cent. solution may be allowed to play on the gums, and at the same time a mouth-wash of 1 per cent. solution should be used frequently. In chronic laryngitis a molecular spray of 1 per cent. solution is found useful. Professor Quincke, of Kiel, is also an enthusiastic advocate of this application, and in nasal diphtheria he recommends a spray of a 1 per cent. solution with 0.75 per cent. sodium chloride added. It has also been found useful in pulmonary gangrene to suppress the fœtid odour of the expectoration.

---

AT a meeting of the Berlin Medical Society Dr. Skaller gave an account of some experiments he has carried out on dogs to determine the effect of Nicotine on Gastric Secretion, not by direct contact of the nicotine with the mucous membrane of the stomach, but by absorption through the blood. He finds that nicotinised animals have a continual gastric hypersecretion, even when fasting, and thinks that it is due to irritation of the peripheral nerve centres by the alkaloid. Experimentally, he finds that atropine is very efficacious as an antidote against this hypersecretion, and he points out that this conforms to clinical observations on man, and that symptoms due to tobacco abuse generally yield to systematic doses of belladonna, provided, of course, the patient at the same time abstains from smoking. Clinically, this gastric hypersecretion may not give rise to any symptoms or may be accompanied by paroxysmal painful sensations in the course of the afternoon or soon after midnight. Finally, the author distinguishes a clinical condition of a more severe type, characterised by actual crises comparable to the crises of tabes dorsalis. This form is accompanied by loss of flesh, and belongs to the group of gastroxynsis of Rossbach, and, in the author's opinion, often fails to be correctly diagnosed.

---

AN account of research, carried out by Drs. Bechhold and Ziegler, to determine the conditions of Solubility of Uric Acid and Sodium Biurate in the blood-serum appears in the *Biochemische Zeitschrift*. The behaviour of these substances in regard to their solubility in such colloidal fluids as blood-serum appears to be complicated, and upon a more perfect knowledge of these conditions depends to a large extent a further elucidation of the phenomena of gout and its allied conditions. When uric acid is left with blood-serum for an hour at 37° C. a certain quantity is dissolved. If the serum is then filtered it is at first clear, but gradually deposits more and more sediment of uric acid. Serum has therefore the property of super-saturation. When heated to 58° C. this property is to a great extent lost. The solubility of uric acid in serum for super-saturation is 1 in 1,000, for saturation 1 in 1,900, whereas the solubility of uric acid in water at 37° is 1 in 15,500. On the other hand, sodium biurate is 60 times less soluble in serum than in water. Dilution of the serum with normal saline and the addition of acid or alkali assists the solution of uric acid or sodium biurate. Potassium, and to a less degree lithium and magnesium hinder precipitation, but sodium, ammonium, and calcium accelerate the precipitation of uric acid. From these facts the authors draw certain therapeutic conclusions. They think that in gouty conditions the ingestion of large quantities of fluid is indicated to dilute the serum and so prevent precipitation of uric acid. Apart from administering the salts of potassium or lithium, a diet of vegetable food containing considerable proportions of these salts should be given. On the other hand, a diet rich in proteids should be avoided, not only on account of the uric acid resulting from their decomposition, but also on account of the ammonium salts, which are seen to accelerate the precipitation of uric acid from the blood.

---

THE relation of Shock to Infection has formed the subject of experimental research by Dr. Gibelli, of Gènes, and an account of the work appears in *La Semaine Médicale*. Clinically, there is a good deal of evidence in support of the view that shock and allied conditions tend to depress the resisting powers of the organism and render it more susceptible to microbic infection. Experimentally, however, the results have not been altogether unequivocal. Galeazzi, for instance, arrived at the conclusion that shock causes a slowing of metabolic changes in the organism, and consequently also the bacteria enter more slowly into the circulation and the toxins are more slowly absorbed. Gibelli produced the condition of shock in guinea pigs, in the same way as Galeazzi, by evisceration and by exposing the viscera to cold. Cultures of B. Anthracis and B. Pyocyaneus were inoculated hypodermically into ten such guinea pigs and ten controls. As a result all the animals submitted to shock died in four to thirty-six hours and before the control animals, but the relation between the number of colonies obtained from the guinea pigs in condition of shock and the controls was not uniform. In four cases there were a greater proportion in the blood from the control, but the reverse was the case in the remaining six. In a further series, however, of similar experiments the author obtained more constant results, and the animals in condition of shock always contained a greater number of bacteria after the same interval of time, and death supervened much earlier. It would appear, therefore, that in conformity with clinical experience the diminished circulatory and metabolic activity of shock and collapse in no way interferes with the invasion of the organism by infective microbes, but that on the contrary such a condition is favourable to infection and renders the organism a more easy prey.

---

In a recent number of the *Centralblatt für Bakteriologie* there appears an interesting article from the pen of Scheller on the dissemination of the Bacillus of Influenza. The author bases his conclusions on the facts he acquired from the study of epidemics of the disease which occurred at Königsberg in 1906-7 and 1907-8. The epidemic of 1906-7 was much the more violent, and the author was able to distinguish the bacillus of Pfeiffer in the sputum in 90 per cent. of cases, and in half of these it occurred in pure culture. In the less serious epidemic of the following winter the bacillus was found in only 20 per cent. of cases: in the remainder the pneumococcus was the organism most frequently met with. During the first epidemic the author performed post-mortem examinations in four cases of influenzal pneumonia, and in all four his findings agreed with Pfeiffer's description of confluent broncho-pneumonia. The exudate from the trachea and larger tubes contained a mixture of Pfeiffer's bacillus with other organisms such as the pneumococcus, streptococcus, and the micrococcus catarrhalis. In proportion as the finer bronchi were examined the more predominant became the bacilli of Pfeiffer, so that in the terminal bronchioles and in the broncho-pneumonic patches these bacilli were found in pure culture. Fibrin was entirely absent. With a view to determining the presence or absence of the bacillus in healthy individuals, the author examined a number of these, and found the organism in 24 per cent. of cases. In the lesser epidemic of 1907-8 this proportion had greatly decreased. From these observations the author is led to conclude that Pfeiffer's bacillus must be considered the specific agent of, at any rate, the more severe epidemics of the disease. In all epidemics a number of cases of influenza occur which are not due to the specific organism, but this number becomes relatively greater when the epidemic is on the decline, whereas at the height of an epidemic there is an overwhelming proportion of cases due to Pfeiffer's bacillus. Sporadic cases resembling influenza, but due to other organisms, may occur apart from any epidemic, but on the other hand the specific bacillus has been found in healthy subjects both during an epidemic and when no outbreak was raging. The tuberculous subject seems capable of retaining the organism for a considerable time without exhibiting symptoms of the disease.

At a recent meeting of the Société Médicale des Hôpitaux, Dr. Sacquépée drew attention to a condition which he termed Acute Cyclic Œdema. This can be distinguished from the acute essential and circumscribed œdemas described under the name of Quincke's disease. Up to the present time acute cyclic œdema has only been observed in the eyelids. From the study of four cases of the affection the author describes its chief characteristics. The phenomena at the outset are those of a general febrile disturbance, followed later by localised pains such as those of trigeminal neuralgia. Next appears the œdema, which is limited to the painful areas, and may be accompanied by pupillary disturbances, ptosis, facial paralysis, and alterations in the reflexes. The malady is of short duration, and no case of a relapse has yet been recorded. Lumbar puncture reveals a lymphocytosis of the cerebrospinal fluid. The author thinks the affection is of an infectious nature, that it may be contagious, and that it is probably accompanied by a slight meningomyelitis or a ganglio-radiculitis. In many respects it resembles herpes zoster.

---

At a meeting of the Société Médicale des Hôpitaux, Ménétrier, Touraine, and Mallet described the results of employing X-rays as Irradiations over the Hepatic Area, in the case of thin patients suffering from severe diabetes. The immediate effect is an exaggeration of the glycosuria, while at the same time in some cases a considerable fall in the number of red-blood corpuscles is found. The remote action is the reverse of the preceding. After a day or two of increased glycosuria the amount of sugar passed in the urine progressively diminishes until it is actually less than before, even where strict diet and opotherapy have failed. Similarly the blood quickly recovers its normal composition, and in a few days, after successive applications of the rays, the red-blood corpuscles are present in quantity above the normal. Results far less marked are met with in stout patients suffering from a mild degree of the affection, it, moreover, being necessary to employ far larger doses of the rays. With small doses inverse phenomena are even noticed. During the interesting discussion which followed Dr. Labbé remarked that the considerable numerical variations in the red-blood corpuscles, without a corresponding modification of the general condition of the patient, could be explained by differences in the dilution of the blood and rearrangement of its constituents. As regards the action of the rays on the glycosuria, he thought that these latter provoked the elimination of sugar in those subjects who were in a condition of hyperglycæmia, and, moreover, the greatest care was necessary in estimating results owing to possible variations in diet.

---

A Surgical Curiosity was exhibited at the Royal Society of Medicine recently by Mr. Ivor Back, and is reported in the *Proceedings* of that body. The patient from whom it was obtained is a girl of eight years old, who had complained of hypogastric pain for eight days, paroxysmal but persistent in character, and beginning in the left half of the abdomen. The bowels were regularly open without aperients, and vomiting had occurred at the onset and also just prior to admission into hospital. The temperature was 97.5° and pulse-rate 106 upon admission, and a hard, tender mass could be felt in the epigastrium, with a distended lower abdomen. Vomiting set in steadily, the pulse-rate increased to 130, and the temperature fell to 96°. At operation the epigastric mass turned out to be an intussusception, whose apex was in the descending colon. After reducing this an oblong body was felt inside the cæcum, and no sign of the appendix could be made out in the usual place. On incising the cæcum the mass proved to be the appendix, the proximal two-thirds of which was involuted into the cæcum and turned inside out; it was acutely inflamed. The distal third twisted back again and lay inside the proximal portion. The specimen was shown in this condition. The explanation suggested was that it was this involuted appendix which had formed the apex of the intussusception; but it was admitted that the sequence of events was obscure and probably unique.

---

In the ordinary way few morbid conditions react so promptly and satisfactorily to treatment as does Scabies. The usual sulphur-ointment cure, with the necessary precautions to prevent reinfection from clothes, has only one drawback, and that is the onset of sulphur dermatitis in some patients. In the *Journal of the Royal Army Medical Corps* are given the observations of Major H. C. French on an alternative line of treatment ordered to be tried at Aldershot in the Cambridge Hospital. On admission a hot bath, with a nail-brush and carbolic soap, was given, and immediately afterwards a mixture of balsam of Peru (four parts) and glycerine (one part) was well rubbed in by an orderly. The next day nothing, not even water was allowed to touch the patient's skin. On the third day the patient was inspected, and allowed to go if no burrows could be seen; otherwise the treatment was repeated. The conclusion arrived at is that if the bath and the sterilisation of clothes are properly carried out any parasiticide will effect a cure. Balsam of Peru costs 10s. a lb., whereas balsam of Gurjun costs but 9d., and sulphur ointment is also very cheap. In mild cases uncomplicated by eczema, impetigo, or furunculosis, liquor calcis sulphurata is better than either. The way to avoid sulphur dermatitis when using sulphur ointment is to apply it once thoroughly, but not to allow any rubbing of it in.

---

A lengthy and elaborate investigation of the whole of the circumstances attending the Involution of the Puerperal Uterus is reported by Dr. Goodall to the *American Journal of Obstetrics*, who sums up in twenty-seven conclusions, the most important and novel of which are the following: The uterus renews all its arteries after each pregnancy; the renewal always consists in the building of a new vessel inside the old one. When a small vessel is thus made inside a very large one (*e.g.* about the placental site) the new vessel has three completely new coats; but if the new vessel is not very much smaller than the old, it forms a new intima only, and takes as much of the old media as is required to complete its wall. The old vessels are in time completely absorbed; the common differences between the parous and ultiparous uterus arise merely out of incomplete destruction. Moreover, the parous uterus can in a young healthy woman return to such a state as to be microscopically indistinguishable from a virgin one; this is, of course, rare. Disease, either local or general, may interfere with this process of involution. The uterine veins are always reduced in size by a growth of hyaline tissue in their walls, and not by organisation of clot. This hyaline tissue is ultimately replaced by muscle, which in the case of the large veins forms new uterine muscular fasciculi. The muscular cells of the uterus and cervix undergo fatty degeneration, with subsequent slow absorption of the fat. The author here, it w[illegible] be noticed, returns to the old views on this subject which prevailed until about twenty years ago, and have since been frequently derided. Many of the muscular fibres of the uterus disappear entirely; this is especially true of the media of the large arteries.

---

The application of some of these facts is to be found in a paper by Dr. Polak, in the same issue, on Some Sequelæ of Labour. This physician, when he examined his puerperal patients on the tenth day after delivery, found retroversion quite exceptional; but when he adopted a routine of examining also between the fourth and sixth weeks he discovered it in nearly 50 per cent. Nearly all of these were easily and successfully treated by a properly fitting pessary, worn until the uterine retraction and involution were completed. The avoidance of lacerations during labour, both of the perineum and also of the cervix, by premature or unnecessary use of the forceps; the prevention of infection of any part of the birth canal, however slight; the complete evacuation of the uterus; and the generous use of ergot until involution is complete and the uterus regains its normal relations to the pelvis, that is, for twelve weeks—these are the means by which post-partum morbidity is to be avoided. As for postural treatment, the author advises that patients should be allowed and urged to change their position frequently. The right and left latero-prone and the prone postures are to be encouraged; the patient should be allowed to sit up in bed for meals and when nursing her child, and to get out of bed for evacuation of the intestines. When the fundus uteri is level with the brim of the pelvis the mother is allowed to sit out of bed, and before returning to recumbence she is placed in the knee-chest position for several minutes. Dr. Polak further commends free hot, sterile douches as soon as the lochia becomes colourless. Perhaps the most unusual of these suggestions is that for the continued use of ergot. If pregnancy, labour, and the puerperium are, as they should theoretically be, physiological processes it is a little odd that such prolonged pharmacological treatment should be thought desirable.

